# Atomic and electronic structure of trilayer graphene/SiC(0001): Evidence of Strong Dependence on Stacking Sequence and charge transfer

**DOI:** 10.1038/srep33487

**Published:** 2016-09-15

**Authors:** Debora Pierucci, Thomas Brumme, Jean-Christophe Girard, Matteo Calandra, Mathieu G. Silly, Fausto Sirotti, Antoine Barbier, Francesco Mauri, Abdelkarim Ouerghi

**Affiliations:** 1 Centre de Nanosciences et de Nanotechnologies, CNRS, Univ. Paris-Sud, Université Paris-Saclay, C2N–Marcoussis, 91460 Marcoussis, France; 2 Institut de Minéralogie, de Physique des Matériaux, et de Cosmochimie, UMR CNRS 7590, Sorbonne Universités, UPMC, Univ. Paris VI, MNHN, IRD, 4 Place Jussieu, 75005 Paris, France; 3 Max Planck Institute for the Structure and Dynamics of Matter, Luruper Chaussee 149, 22761 Hamburg, Germany; 4 Synchrotron-SOLEIL, Saint-Aubin, BP48, F91192 Gif sur Yvette Cedex, France; 5 Service de Physique de l’Etat Condensé, DSM/IRAMIS/SPEC, CEA-CNRS UMR 3680, CEA-Saclay, F-91191 Gif-sur-Yvette, France; 6 Departimento di Fisica, Università di Roma La Sapienza, Piazzale Aldo Moro 5, I-00185 Roma, Italy; 7 Graphene Labs, Fondazione Istituto Italiano di Tecnologia, Via Morego, I-16163 Genova, Italy

## Abstract

The transport properties of few-layer graphene are the directly result of a peculiar band structure near the Dirac point. Here, for epitaxial graphene grown on SiC, we determine the effect of charge transfer from the SiC substrate on the local density of states (LDOS) of trilayer graphene using scaning tunneling microscopy/spectroscopy and angle resolved photoemission spectroscopy (ARPES). Different spectra are observed and are attributed to the existence of two stable polytypes of trilayer: Bernal (ABA) and rhomboedreal (ABC) staking. Their electronic properties strongly depend on the charge transfer from the substrate. We show that the LDOS of ABC stacking shows an additional peak located above the Dirac point in comparison with the LDOS of ABA stacking. The observed LDOS features, reflecting the underlying symmetry of the two polytypes, were reproduced by explicit calculations within density functional theory (DFT) including the charge transfer from the substrate. These findings demonstrate the pronounced effect of stacking order and charge transfer on the electronic structure of trilayer or few layer graphene. Our approach represents a significant step toward understand the electronic properties of graphene layer under electrical field.

The peculiar band structure features of massive Dirac fermions in multilayer graphene are driving intense activity in fundamental research, as well as for applications in next generation optoelectronic devices^1^. The electronic properties of graphene layers depend on the in-plane and out-of plane arrangement of their carbon atoms in the atomic distribution, it can be described using edge/crystal orientation of graphene planes and stacking orders^2^. The influence of the edge orientation on the physical properties of graphene flakes and nanoribbons has been predicted theoretically and measured experimentally^3^.

In few-layer graphene (FLG), the interlayer coupling introduces perturbations of the low-energy band dispersions. Consequently, the linear *π* and π* bands near the Fermi level in monolayer graphene are modified in FLG, showing strong dependence on the stacking sequence as well as the number of layer^4^,^5^. In analogy with what observed in multilayer graphene, the three possible types are: AA stacking (simple hexagonal, space group of *P*6/*mmm*), ABA stacking (hexagonal, called Bernal, *P*63/*mmc*), and ABC stacking (rhomboedrical, *R*1/3*m*)^6^. Ambipolar transport has been demonstrated experimentally in the Bernal stacking while an electric-field-induced band-gap opening was observed in the AB-bilayer^7^. Thus, both determination and control of the stacking sequence are necessary for graphene-based electronics. For trilayer graphene a mixture of both ABC stacking and ABA stacking has been observed using ARPES^8^,^9^. Recently, other experimental studies have revealed that ABC trilayer graphene, as identified by a peak asymmetry in micro-Raman spectroscopy, has different electrical properties compared to ABA stacking trilayer graphene^10^.

Among the different methods to obtain few layer graphene, SiC graphitization is a viable method for a large-scale graphene production^11^,^12^. In particular, in the case of Si-face, the Si sublimation during the graphitization process results in a C-rich 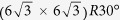 surface reconstruction of the SiC surface. The presence of this buffer layer is very important because it provides a hexagonal template for the epitaxial graphene growth which induces a layer by layer growth and facilitates long-range order in the epitaxial graphene with a precise stacking (Bernal or rhombohedral). The growth on SiC underlayer has been developed and used to produce high quality wafer scale monolayer graphene^13^. However only few papers discuss the electronic properties of the few layer epitaxial graphene (N ~ 2–4) on SiC(0001)^14^,^15^. This epitaxial graphene presents an intrinsic n-type doping. This doping is explained conventionally by donor-like states associated with the buffer layer and its interface to the substrate that overcompensate the polarization doping due to the SiC substrate^16^,^17^. However, this charge transfer from the substrate to the graphene layer and its influence on the electronic properties of few layer graphene remain unexplored until now. In this regard, scanning tunneling microscopy (STM) and spectroscopy (STS) are powerful tools to study the local structural and electronic properties of graphene down to atomic scale^8^,^15^,^18^,^19^,^20^,^21^,^22^.

Here, large-area uniform trilayer graphene layers were successfully synthesized on off-axis 4H-SiC(0001). The morphology of the trilayer graphene consisting of large and flat domain was confirmed by LEEM. The band structure and the charge transfer from the substrate to graphene layer were studied using ARPES. The electronic properties and the stacking order trilayer graphene are studied by STM/STS and density functional theory (DFT). Our studies indicate that trilayer epitaxial graphene exhibits two structure with Bernal (Fig. 1(a)) and rhombohedral (Fig. 1(b)) stacking (ABA and ABC respectively). By exploiting STM/STS we examine the electronic structure of ABA and ABC trilayer graphene and compared it to DFT calculations, explicitly taking into account the charge transfer from the substrate and the resulting asymmetric electric field. Note that this level dependence cannot be modeled by a rigid shift of the Fermi energy or the commonly used homogeneous background doping method.

## Results and Discussion

**Figure 2:**
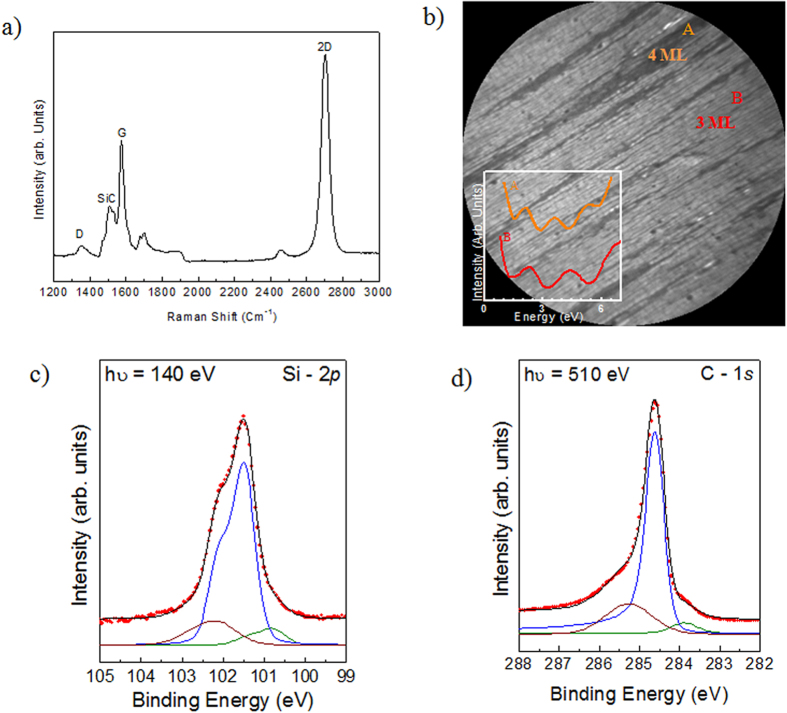
Morphological, micro-Raman and XPS of graphene/SiC(0001). (**a**) Micro-Raman spectrum showing the characteristic G and 2D bands for the trilayer graphene, (**b**) bright-field LEEM image of epitaxial graphene (LEEM 20 × 20 mm^2^), the electron reflectivity spectra obtained for the region A (4 ML) and B (3 ML) are plotted in inset, (**c**,**d**) C *1s* and Si 2p XPS spectra for epitaxial graphene obtained with a photon energy of 140 eV and 510 eV, respectively.

The trilayer graphene used in this study was obtained by annealing 4° off-axis 4H-SiC(0001) at 1550 °C in 800 mbar argon for 10 min. We used micro-Raman spectroscopy to probe the quality and electronic properties of the sample. Figure 2(a) shows a representative Raman spectrum of a graphene/SiC system in the wavelength range 1200–3000 cm^−1^. The peak at 1522 cm^−1^ is considered to be an overtone of the L-point optical phonon from the SiC. The Raman signals from the graphene show prominent characteristic peaks at 1595 cm^−1^ (G) and 2705 cm^−1^ (2D), which gives evidence of carbon *sp*^2^ reorganization. The G peak value of 1595 cm^−1^ indicates n type doping. Also the blue-shifted position of the 2D peak at 2705 cm^−1^ could be the result of a compressive strain of the graphene layer during the post-growth cooling down procedure or charge transfer doping from the substrate^23^. This blue shift of the G and 2D band has been also reported in epitaxial graphene on 6H-SiC wafer^8^. The 2D peak is however much broader than in isolated graphene with a full width at half maximum (*fwhm*) of about 75 cm^−1^. This broadening can be attributed both to a defect scattering and to the formation of two or more graphene layers. The intensity ratio of the defect-induced D band to the graphite G band (I_D_/I_G_) has been widely used to estimate the density of defects in graphene^16^, and also the graphene crystallite size (*La*)^24^. In fact using the following equation 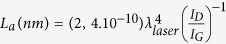 (Where λ_*laser*_ is the Raman excitation wavelength (λ_*laser*_ = 532*nm*) and the measured ratio 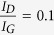 we obtained a crystalline size of about of 600 nm. Moreover, the low D over G ratio demonstrates the good structural quality of the graphene layers. The graphene sample was further characterized by LEEM microscopy in order to evaluate the thickness spatial distribution. Figure 2(b) shows typical Bright-Field (BF) LEEM image pattern from graphene layer of our sample. The electron energy is 1.2 eV and a field of view (FOV) of 15 *μ*m. The regions with two different graphene thickness can be distinguished by differences in the reflected intensity^25^,^26^, as shown in the inset of Fig. 2(b). Although surface domains with grayscale contrasts can be identified, the sample is highly homogeneous with the light-gray domains (label (B)) occupying more than 90% of the overall area. Similar pictures were taken over an extended electron energy range to describe the electron reflectivity. The number of dips in the electron reflectivity spectra (insert of Fig. 2(b)) confirms that these areas consist of trilayer while the small regions with medium-gray (label (A)) contrast are tetralayer graphene. Some sharp dark lines, which are all aligned along the < 1–100 > SiC axis, start to appear on the surface, showing that the graphene layer nucleates on the inter-terrace nanofacets. Typically these ribbons are 600 nm wide and ~500 μm long and are all aligned along the < 1–100 > SiC axis, as it has already been observed on the monolayer graphene on off-axis SiC(0001) surface^27^.

The chemical bonding environment of the sample has been probed using X-ray Photoelectron Spectroscopy (XPS) experiments. High-resolution spectra of Si 2p and C 1 s recorded under surface sensitive conditions (hv = 140 eV and 510 eV, respectively) are shown in Fig. 2(c,d), respectively. Due to the low electron inelastic mean free path at these photon energies^11^, only the 2–5 first topmost layers were probed. The different components contributing to the spectra were deconvoluted using a decomposed in individual lines through a curve fitting procedure. The experimental data points are displayed with dots, meanwhile the red solid line is the envelope of the fitted components. A Shirley background was used and Fig. 2(c) shows the subtracted Si 2*p* spectrum. The spectrum was fitted by sums of Voigt curves (i.e, the convolution of a Gaussian by a Lorentzian, black line). The Lorentzian width (*fwhm*) was fixed at 45 meV^28^. The Si 2*p* peaks are made up of spin-orbit split doublets (2p_1/2_:2p_3/2_ ratio of 0.5 and a spin-orbit splitting of 0.6 eV^29^), and the binding energies are given with respect to the Si 2*p*_3/2_ position. The main peak at 101.5 eV corresponds to the SiC bulk contribution, the small shoulder at 102.1 eV is attributed to the interface layer area of the SiC(0001) substrate while the component at 100.9 eV is attributed to the presence of Si clusters formed when Si-C bonds are broken during graphitization^30^,^31^. Figure 2(d) shows the C 1 s peak. After a Shirley background subtraction the C 1s spectrum was fitted by a sum of a Gaussian function convoluted with a Doniach-Sunjic lineshape. The C *1s* spectrum showed three components at 283.4, 284.5, and 285.3 eV in binding energy. The sharp C *1s* peak, labeled G, located at 284.5 eV in binding energy, indicates the presence of sp^2^ hybridized C–C bonds and is the signature of the graphene layers. Due to its metallic nature this component was fitted using a Doniach-Sunjic line shape with an asymmetry factor α of 0.1 and a *fwhm* of 0.5 eV. The two other peaks labelled SiC (283.4 eV) and IL (285.3 eV) are attributed to the SiC bulk and to the interfacial layer respectively. Due to the non-metallic nature of these components, the peaks are symmetric (asymmetric factor α = 0 eV). From the C 1 s spectrum, the thickness of the graphene layers can be calculated from the ratio between the intensities of the G and SiC components. This ratio corresponded to an exponential decay of roughly 3 ML of carbon coverage supposing a continuous and homogenous coverage of the surface^27^.

**Figure 3:**
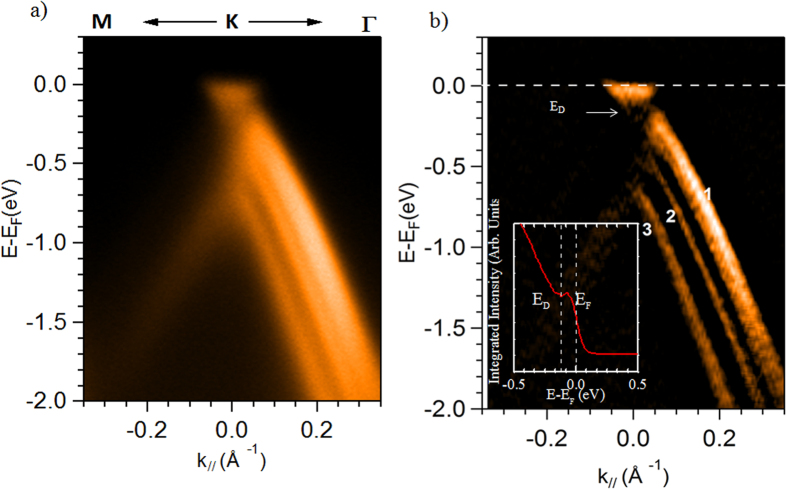
Electronic structure of multilayer graphene. (**a**) Angle resolved photoemission spectrum of epitaxial graphene/SiC(0001), revealing the 3 bands structure, signature of trilayer graphene. The spectrum is measured with photon energy of 60 eV and with scans oriented along the ΓK direction of the graphene Brillouin zone, (**b**) Second derivative of the intensity ARPES data along the ΓK direction of the trilayer graphene, the position of the Dirac point (about 150–200 meV below the Fermi level, see integrated spectrum inset of Fig. 3(b)), is highlighted by a white arrow.

The electronic structure was also probed using ARPES, which gives direct access to the spectral function containing the information on electron energy band dispersion. The measured ARPES band structure map is displayed in Fig. 3(a), in which the photoelectron intensity is represented as a function of energy and k-momentum, along to the Γ–K direction of the graphene first Brillouin zone^32^. In Fig. 3(b) the second derivative spectrum of the ARPES map of Fig. 3(a) is provided to improve the band structure visibility. Three sharp and intense π-bands are visible on the spectra confirming the high structural quality of the graphene layers. An increased splitting is observed between the second and third band indicating a higher contribution of the ABA stacking^4^ with respect to the ABC one. Moreover, the large dispersion of the first band indicates that also a small contribution of tetralayer is present on the sample as observed with LEEM (Fig. 2(b)). From the ARPES map we can also give an approximate estimation of the energy distance between E_F_ and E_D_ (see also the integrated spectrum extracted from the ARPES map of Fig. 3(a) in the inset of Fig. 3(b)), which is in the range of 150 to 200 meV. This is the value expected for epitaxial graphene, as the interface layer acts as dopant for the graphene/SiC(0001). From a linear fit, using the relation E = *hv*_F_*k*, we obtain the value of the Fermi velocity *v*_*F*_ = 1.06×10^6^ m/s, which matches the expected value using DFT theory^4^.

**Figure 4:**
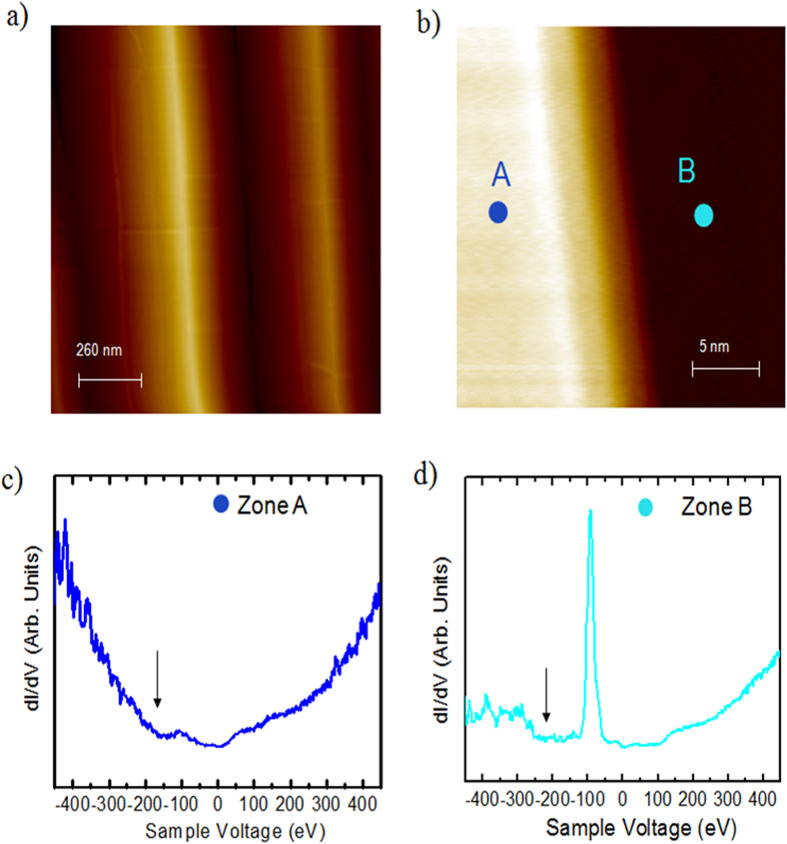
STM/STS studies of trilayer epitaxial graphene (T = 4.2 K). (**a**) Large scale STM topographic image (1000 × 1000 nm^2^, 800 meV, 0.5 nA), (**b**) STM image of trilayer graphene over a terrace (25 × 25 nm^2^, 100 mV, 200 pA), (**c**) STS spectrum acquired in the zone A, left side of the figure (**b**,**d**) STS spectrum acquired in the zone B, right side of figure (**b**). The local minimum in the *dI/dV* curves highlighted by the arrow indicate the position of the Dirac point.

**Figure 5:**
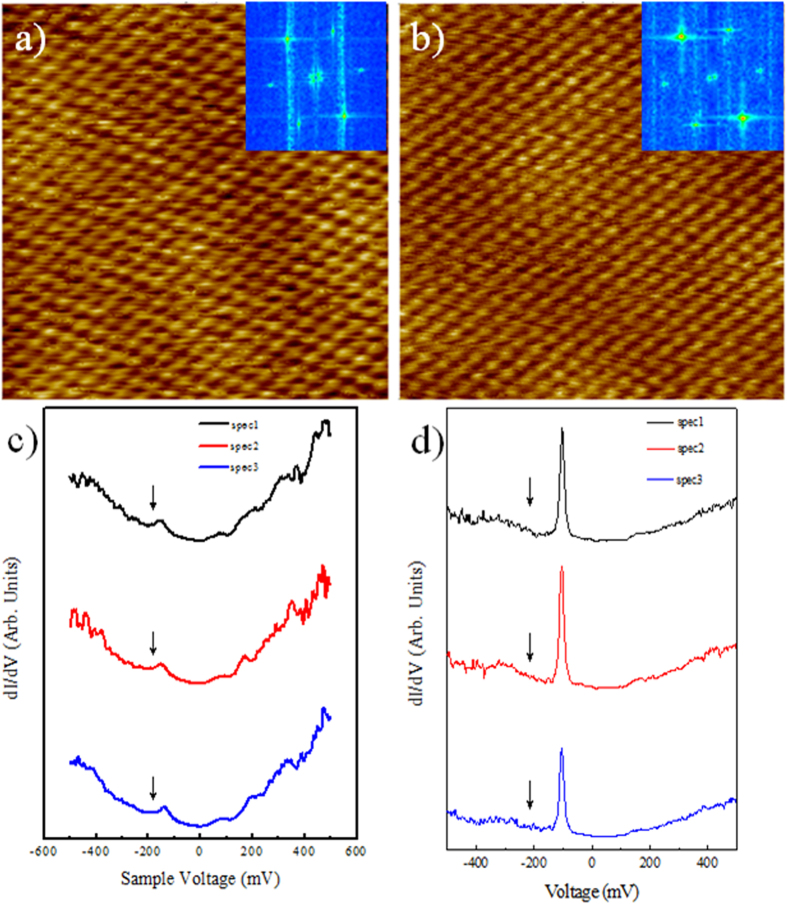
STM/STS studies of trilayer epitaxial graphene (T = 4.2 K) with atomic resolution. (**a,b**) Typical atomic resolution STM images of our sample (5 × 5 nm^2^, 100 mV, 200 pA). In insert the Fourier Transform image of the two phases, (**c**,**d**) STS spectra obtained in different positions of figure (**a,b**), respectively. The local minimum in the *dI/dV* curves highlighted by the arrow indicate the position of the Dirac point.

STM was performed within the terrace regions in order to study the electronic properties of the trilayer graphene/SiC(0001) surface. The surface is formed by self-ordered periodic structures consisting of pairs of a (0001) basal plane terrace and a (1–10n) nanofacet with a characteristic periodicity of ∼600 nm, as shown in Fig. 4(a). This surface shows very flat terraces (Fig. 4(b)) separated by fairly straight step edges. The triangular lattice structure is resolved on both upper and lower terraces with the lattice orientation remaining unchanged. Moreover, the triangular lattice structure of the graphene overlayer remains perfect over the step, which further demonstrates that the graphene is continuous over the step edge. Thanks to STS measurements the local electronic structure of the sample was probed. Figure 4(c,d) display the STS spectra recorded in the upper (zone A, left side) and lower part of the terrace (zone B, right side) of Fig. 4(b). The local minimum in the *dI/dV* curves highlighted by the arrow indicate the position of the Dirac point which is shifted with respect to the Fermi energy due to the charge transfer between the SiC substrate and the graphene layer. A standard V-shaped spectrum is evident in the left region (zone A, Fig. 4(c)) of Fig. 4(b), no gap like feature is present in the spectrum^18^,^19^,^20^,^21^,^22^. A sharp peak in the *dI/dV* located at ~ −100 meV is clearly resolved in the spectrum measured in the right region (zone B, Fig. 4(d)). The difference between the two spectra demonstrates that the trilayer graphene/SiC is heterogeneous in electronic structures, which are typically at least a few nanometers in size. Figure 5(a,b) shows two typical STM images of the graphene layers with an atomic-resolution, taken with a sample tunneling bias V = 50 meV in two different regions of the sample. The long range periodicity of the surface is easily observed. The hexagonal arrangement within the carbon layer and its orientation remains undisturbed as confirmed by the nearly identical Fourier Transform image of the two phases (insert Fig. 5(a,b)). From the FTs, we obtain a spatial periodicity of 2.5 Å for the two surfaces. No difference in lattice constant between the two regions is resolved, indicating that the graphene is largely strain-free. Figure 5(c,d) display the corresponding STS spectra of the Fig. 5(a,b) respectively.

**Figure 6:**
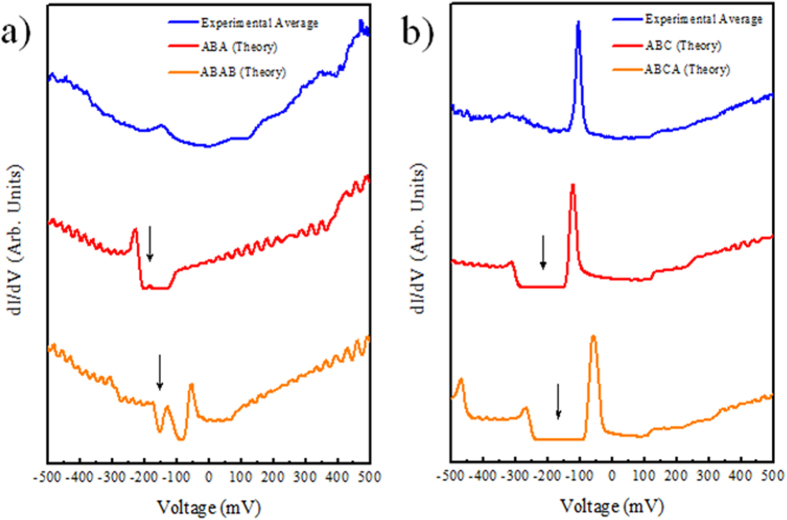
Experimental *dI* /*dV* curves for trilayer graphene together with the calculated LDOS. (**a**) Experimental *vs* theoretical STS spectra of three and four layer Bernal stacking, and (**b**) Experimental *vs* theoretical STS spectra of three and four layers rhombohedral stacking. The local minimum in the *dI/dV* curves highlighted by the arrow indicate the position of the Dirac point.

In order to understand how the tunneling spectra depend on the stacking of the layer we compare the STS results with the local density of states (LDOS) as calculated by DFT. This comparison reported in Fig. 6(a,b) allows for the discriminations between the Bernal and rhombohedral stacking. The interpretation of tunneling spectra of the combined FLG/SiC(0001) system in terms of the graphene band structure may be somewhat difficult because of the underlying 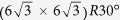 interface layer, which can contribute to the tunneling current. However, the contribution of the interface layer to the tunneling current is expected to exponentially decay with the thickness of the few layer graphene so that the undistorted electronic structure of the films will become observable for multilayers, as in the present case. To capture the main features observed in the STS spectrum, we included in the model only the charge transfer from the substrate to the graphene layer. In order to correctly describe the doping due to the SiC substrate and the resulting asymmetric electric field we used our method for the calculation of the field effect setup from first principles^33^ in order to correctly describe the decreasing charge carrier concentration per layer found in experiments^34^. Note that this level dependence cannot be modeled by a rigid shift of the Fermi energy or the commonly used homogeneous background doping method. We doped the trilayer graphene with 7.7 × 10^−3^ electrons per unit cell^34^ as the ARPES measurements showed that the Dirac point is shifted to E_D_ in the 150–200 meV range. Figure 6 shows the calculated LDOS for the ABA and ABC trilayer graphene (Fig. 6(a)) and ABAB and ABAC tetralayer graphene (Fig. 6(b)). The calculated LDOS is the sum of the DOS projected onto the p_z_ orbitals of the uppermost graphene layer. That this is a reasonable approximation follows from the fact that the STM signal is proportional to the LDOS integrated between the Fermi energy and the corresponding bias voltage^35^. Thus, the STS will be proportional to the LDOS at the bias voltage. In graphene the bands close to the Fermi energy are composed of the p_z_ orbitals and since the p_z_ orbitals of the uppermost layer of the multilayer sample have the largest extend into the vacuum region they will contribute most to the signal. We also verified this for the ABC trilayer by calculating the numerical derivative of the STM signal as obtained with the Tersoff-Hamann approximation^35^ taking into account all states. This results in essentially the same curves with minor changes in the relative height of the peak at −0.1 V and the other peaks. The comparison between spatially *dI/dV* versus bias (V) spectra and the calculated LDOS shown in Fig. 6 permitted us to identify the Bernal or rhombohedral stacking and the thickness of the sample. The presence of a sharp peak in the calculated LDOS, suggests that the STS spectra in Fig. 6(b) can probably be identified as rombohedral stacked multilayer graphene (ABC). Even if the situation is less clear for the spectra in Fig. 6(a) as the agreement theory/experiment is worse, the absence of a sharp peak at the Fermi level exclude rombohedral stacking in these area. We underline that with the increase of the number of layer, *e.g* in the case of ABCA stacking, the variation in charge transfer induces different intensity ratio between the peak above the Dirac point and the secondary peaks at 200–300 meV.

As compared to the Bernal stacking, the rhombohedral stacked few layer graphene exhibits band dispersions near the Dirac point. Specifically, the low-energy bands near the Dirac level (flat band) in ABC-stacked few layers graphene has a surface states with its wave functions distributed on either *α* or *β* atoms of the outermost layers. The DFT studies taking into account the doping of graphene due to the interaction with the SiC underlayer are able to reproduce the observed STS results which are different from the case of undoped rhombohedral stacked graphene. The present analysis is only possible because the STM/STS are very sensitive to the stacking, and because of the very high energy of the STM/STS experiment. Note that the total energy difference between *ABA* and *ABC* stacked trilayer graphene is very small (0.18 meV/atom)^36^, so that the presence of both configurations is expected.

## Conclusions

In summary, we identify the three bands due to interlayer interactions and two stacking sequences of the trilayer graphene using ARPES and STM/STS measurements. We find that the stacking and the charge transfer change significantly the LDOS of the few layer graphene, which illustrate the unique electronic properties of graphene layers. The STS and DFT spectra show a clear evidence for flat bands near Dirac energy in trilayer graphene on SiC. The flat bands, which contribute to a pronounced peak in the tunneling density of states, arise from ABC stacking. The morphology of the trilayer graphene consisting of large and flat domain was confirmed by LEEM. The lateral domain size is only limited by the step edges pre-existing in the substrate. STS showed the presence of epitaxial trilayer graphene with a two- stacking domain arrangement of carbon atoms.

## Methods

The few layers graphene were synthesized on off axis *n*-type 4H-SiC(0001) substrates that were etched in hydrogen at 1550 °C and annealed at 1525 °C in argon partial pressure of P = 800 mbar. The samples were then cooled down to room temperature and transferred *ex-situ* from the growth chamber to undergo STM/STS, XPS/ARPES measurements^12^.

STM/STS measurements were carried out using an Omicron ultra-high vacuum low temperature scanning tunnelling microscope (UHV-LT-STM)^37^. The STM tips used in this study were electrochemically-etched polycrystalline tungsten tips, flashed by Joule heating under UHV to temperatures up to white light emission followed by field emission. This procedure allowed to estimate the tip apex radius but also to control the stability of the emission current relative to the quality and cleanness of the tip. In addition during STM/STS experiments, the spectroscopic behaviour of the tip was systematically checked on a clean Au(111) surface in order to ensure that the tips were free of adventitious carbon contaminations. For the STS measurements, performed at *T* = 4.2 K, the I(V) characteristics were acquired while the feedback loop was inactive, the differential conductivity dI/dV (V, x, y), proportional to the LDOS, was measured directly by using a lock-in technique. For this purpose a small AC modulation voltage *V*_mod_ was added to *V (V*_mod, pk-pk_ = 10 mV, *f*_mod_ = 973 Hz) and the signal *dI* detected by the lock-in amplifier was used to determine the differential conductivity *dI* /*dV*_mod_.

XPS/ARPES measurements were conducted at the TEMPO beamline of the SOLEIL synchrotron (Saint-Aubin, France)^38^. The analyzing chamber was equipped with a SCIENTA-2000 electron hemispherical analyzer with a delay-line 2D detector, which optimized the detection linearity and signal/background ratio. The samples were cooled to 120 K by liquid nitrogen. For the ARPES measurements the photon energy was 60 eV with the overall energy resolution of 20 meV.

The electronic structure calculations were performed using the QUANTUM-ESPRESSO code^39^. We used the local density approximation, norm-conserving pseudopotentials and a plane wave energy cutoff of 65 Ry. For the electronic integration in the self-consistent calculation, we used a 256^2^ electron-momentum mesh with an 1 mRy Methfessel-Paxton first-order spreading^40^. We computed the local DOS using a denser 512^2^ electron-momentum grid. In all figures of the paper, the DFT band energy was rescaled by a 1.18 multiplicative factor to correct for the DFT underestimation of the Fermi velocity^41^. The doping due to the SiC substrate was modeled following ref. 33. The FLG system was separated from the charged plate simulating the substrate by a 3 Å thick and 1 Ry high potential barrier to avoid charge spilling into the vacuum.

## Additional Information

**How to cite this article**: Pierucci, D. *et al.* Atomic and electronic structure of trilayer graphene/SiC(0001): Evidence of Strong Dependence on Stacking Sequence and charge transfer. *Sci. Rep.* **6**, 33487; doi: 10.1038/srep33487 (2016).

## Figures and Tables

**Figure 1:**
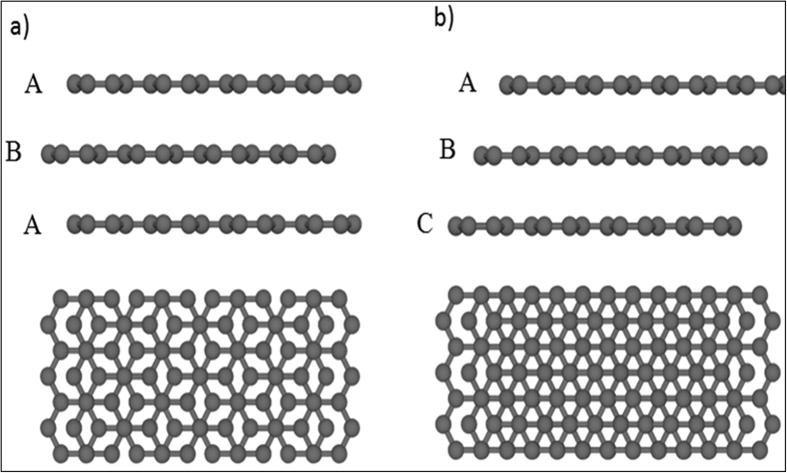
Structure of trilayer graphene with (**a**) Bernal ABA and (**b**) rhombohedral ABC stacking sequence, respectively: the dots represent the A and B sublattices of the graphene honeycomb structure.
